# Liver Transplantation in an Adult with Citrullinaemia Type 2

**DOI:** 10.1155/2011/176370

**Published:** 2011-05-10

**Authors:** Hui-Hui Tan, Wan-Cheng Chow, Kiat-Hon Lim, Wei-Keat Wan, Alexander Y. F. Chung, Peng-Chung Cheow, Chee-Kiat Tan

**Affiliations:** ^1^Liver Transplant Service, Department of Gastroenterology and Hepatology, Singapore General Hospital, Singapore 169608; ^2^Liver Transplant Service, Department of Pathology, Singapore General Hospital, Singapore 169608; ^3^Liver Transplant Service, Department of General Surgery, Singapore General Hospital, Singapore 169608

## Abstract

Citrullinaemia is a urea cycle defect that results from a deficiency of the enzyme arginosuccinate synthetase. Type 1 disease is diagnosed in childhood, whereas Type 2 disease is adult onset. We report the outcome of a patient with citrullinemia Type 2 who received a liver transplant at our center and the implications of this diagnosis in liver transplantation.

## 1. Introduction

Citrullinaemia is a urea cycle defect that results from a deficiency of the enzyme arginosuccinate synthetase. Type 1 disease is diagnosed in childhood, whereas Type 2 disease is adult onset. We report the outcome of a patient with citrullinemia.

## 2. Case Report

**Figure 1:**
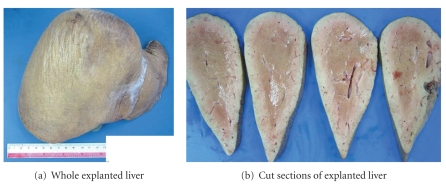
Gross specimen of explanted liver.

**Figure 2:**
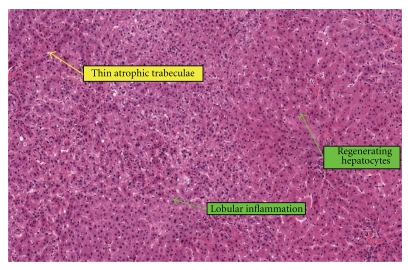
Histomicrograph of explanted liver.

Our patient was 25 years old when she migrated to our country from China. Her medical and childhood history was scant although both the patient and her mother insist these were uneventful and that she had been an average student in school. The patient presented to our hospital at the age of 28 years old for seizures and altered behaviour. She was described by her husband to be responding slowly when spoken to. These episodes were interspersed by verbal and physical aggression, violent outbursts, or hypersomnolence (sleeping for >24 hours) with urinary incontinence. Computed tomography (CT) and magnetic resonance imaging (MRI) of the head were normal. Electroencephalography performed during episodes of hypersomnolence was compatible with global moderate diffuse encephalopathy. Arterial ammonia (195 mmol/L) and lactate (4.5 mmol/L) levels were markedly elevated. However, other liver tests and imaging were not suggestive of liver cirrhosis and excluded the presence of any portosystemic shunt. CT imaging revealed an atrophic pancreas. Tests for hepatitis B, hepatitis C, HIV, Wilson disease, systemic autoimmune disease, and syphilis were negative. In view of her significant behavioural change and elevated ammonia levels, she was screened for inborn errors of metabolism. Citrulline levels in the blood (939 umol/L) and urine (19.6 umol/mmol) were elevated 15-fold and 5-fold, respectively. There was corresponding elevated plasma arginine and threonine : serine ratios. She was commenced on L-arginine 1 g QDS and high-dose lactulose therapy which reduced the frequency of her violent outbursts and reduced the hypersomnolent episodes to once per fortnight. However, these episodes became increasingly difficult to control despite L-arginine replacement. By 2009, she was encephalopathic/hypersomnolent as often as 3 to 4 times per week. The frequency and nature of these episodes also made drug compliance difficult and affected her daily activities. Orthotopic liver transplantation (LT) from a deceased donor was performed in January 2010. The explanted liver was slightly enlarged measuring 21 × 16 × 7.5 cm and weighed 1.036 kg. The gross cut sections appeared pale brown, and the liver emitted a peculiar sweetish odour (Figure 1). Histology showed regenerative changes with alternating atrophied and hyperplastic liver plates. Mild lobular inflammation with ballooning change in the hepatocytes was demonstrated. No significant steatosis or fibrosis was seen (Figure 2). Immunosuppression consisted of prednisolone, tacrolimus, and mycophenolate mofetil. She has recovered well after LT, without further episodes of encephalopathy/hypersomnolence despite a normal protein diet. Arterial ammonia levels (23 umol/L) normalised less than 2 weeks after LT. As her sensorium and ammonia levels had returned to normal and she was able to function in the mental capacity of a normal healthy person, citrulline levels and excretion were not measured again after transplant. She is currently on tailing doses of dual immunosuppression (tacrolimus and mycophenolate mofetil). She continues to remain well more than 1 year after LT and has since returned to work.

## 3. Discussion

Citrullinaemia is a urea cycle defect that results from a deficiency of the enzyme arginosuccinate synthetase (ASS). Nitrogen from enteral sources (dietary protein) and muscle is excreted from the body, as urea, via the urea cycle. Two moles of nitrogen (1 from ammonia, 1 from aspartate) are converted to urea in each cycle. ASS converts citrulline to arginosuccinic acid in the urea cycle, removing ammonia in the process. Deficiency of ASS results in citrullinaemia, an autosomal recessive disorder. 

The severity of disease and clinical presentation is proportionate to residual enzyme activity, dietary protein load, and patient age. 

### 3.1. Citrullinaemia Type I (CTLN1)

Is the classical form of ASS deficiency, where ASS is deficient in all tissues. In severe forms, newborns present with hyperammonaemia, vomiting, feeding difficulties, and seizures soon after birth. In milder disease, patients may present later with mild clinical symptoms or remain asymptomatic in the early neonatal period or infancy. Untreated newborns or infants universally develop cerebral oedema. Morbidity and mortality are high. In one study of newborns, although the 10-year survival rate was 72%, survivors had mental retardation and growth impairments [1].

### 3.2. Citrullinaemia Type 2 (CTLN2)

Is adult onset, and ASS deficiency is limited to the liver [2]. Patients with Type 2 disease usually present between the ages of 20 to 50 years although patients younger or much older than this have also been reported. Type 2 disease is believed to be caused by mutations in the gene (SLC25A13) which encodes citrin, a mitochondrial aspartate glutamate carrier protein (AGC2), located on chromosome 7q21 [3, 4]. Most reports of Type 2 disease have been from Japan, where the incidence is described to be 1 in 100,000 [3]. Individuals from other countries with mutations different from the common Japanese mutations have been identified such as Israel, North America, United Kingdom, Czech Republic, and Pakistan [5–10]. Patients are described to be thin, with up to 40% having a body mass index under 17 and tend to have a predilection for protein-rich foods [11]. Liver dysfunction is minimal or absent. Most patients present suddenly with hyperammonemia and associated neuropsychiatric symptoms such as altered conscious levels, irritability, seizures, or coma. A link between Type 2 disease and chronic pancreatitis has also been described [12–14]. Cerebral oedema is the commonest mode of death and usually occurs several years after onset. 

Management of the disease involves strategies to remove ammonia, maintenance of nitrogen excretion, reducing the frequency of intercurrent episodes and nutritional and fluid repletion. Although patients with urea cycle defects are commonly treated with low protein-high carbohydrate diets, this is harmful in patients with Type 2 citrullinaemia. High carbohydrate intake increases NADH production, further disrupting the urea cycle and stimulating the citrate-malate shuttle, resulting in further hyperammonemia, hypertriglyceridaemia, and fatty liver disease [15–18]. 

Presently, only LT is curative of the disorder [19, 20]. With successful LT, hyperammonemic episodes cease, dietary restrictions are not required, and alternative pathway medication can be discontinued [21–26]. Successful LT for Type 2 disease has been reported with both deceased donor and living donor grafts. Auxiliary partial orthotopic LT has also been described for the treatment of urea cycle defects. Such transplants using a left lobe graft have been described to provide sufficient enzyme supplementation and can correct citrullinaemia [27]. However, this technique too has its complications, such as competition of blood inflow between the native liver remnant and the graft and higher morbidity rates compared to nonauxiliary transplanted recipients [28, 29]. Unlike in Type 1 disease, the metabolic correction of type 2 disease with liver transplantation is complete, as the enzyme deficiency is limited to the liver. Optimum timing for LT is unknown although the aim is to correct the underlying metabolic error before irreversible brain damage occurs. Ikeda et al. reported that 1 of 7 citrullinemia Type 2 patients who underwent living-related liver transplantation in their series continued to be cognitively impaired despite transplantation, whereas the rest had recovered completely from their neuropsychiatric symptoms [30]. This patient had CT and MRI evidence of diffuse cortical atrophy of the brain and marked decreased radionuclide uptake on brain-spectroscopy images. A review based on worldwide data of LT for urea cycle disorders suggested that neurological impairments were more likely to persist after LT in paediatric rather than adult patients and in patients receiving a deceased donor graft rather than a living donor graft [25]. Some authors advocate consideration for transplantation as soon as the first presentation of metabolic decompensation [9].

The true incidence of urea cycle defects is not known as the disorder is commonly missed. The presentation of neonates or infants is nonspecific and most paediatric patients are evaluated for sepsis or respiratory disorders instead [19]. Unless specifically tested for, hyperammonaemia and an evaluation for its cause may be missed in the diagnosis. The same is true in adults. This has specific implications for liver transplantation. Firstly, organs from deceased donors should not be accepted if the aetiology of the cause of death is vague. Plochl et al. reported an unfortunate patient who developed acute hyperammoanemic encephalopathy and died after receiving a liver from a male donor who had died of atraumatic cerebral oedema of unknown cause [31]. Ornithine transcarbamylase deficiency was subsequently diagnosed from the measurement of urea cycle enzymes and molecular analysis of donor liver tissue. The maternal uncle of this donor had also died from coma of unknown cause. To our knowledge, no such incident has been reported of a deceased liver donor with undiagnosed citrullinaemia although it would be logical to assume that the same poor outcome could happen in such a situation. Secondly, deceased donor grafts are scarce in many parts of the world, necessitating living donor liver transplantation instead. As living donor grafts are usually harvested from relatives, a thorough donor workup should be undertaken to exclude an ASS deficiency state in the donor, since this is an inheritable disorder. Apart from measuring plasma citrulline and ammonia levels, some centres also biopsy donor liver tissue to measure urea cycle enzymatic activity [25, 30]. In the series by Morioka et al., parent donors were accepted if their plasma ammonia levels and quantitative serum amino acid analyses were normal despite their heterozygosity for CTLN2 [25]. However, a sibling donor was further evaluated with a liver biopsy and hepatic enzymes were assayed despite normal plasma ammonia and quantitative serum amino acid analysis in view of the nature of inheritance of this disorder. Based on hepatic enzyme assay, this sibling donor was either heterozygote or latently diseased. However, in view of the need for an emergent transplant and his being the only available donor, a left liver auxiliary partial orthotopic transplant was performed with informed consent. Both donor and recipient remained well beyond 77 months from liver transplantation. The use of asymptomatic heterozygote donors has not been reported to be associated with any significant morbidity or mortality thus far although their use is yet to be validated or established. 

Hepatocyte transplants and gene transfers have been explored for the treatment of urea cycle defects, including citrullinaemia. However, these are not without risks, and their current role remains in a research setting.
